# Formulation Development and Optimization of Fast Dissolving Tablets of Aceclofenac Using Natural Superdisintegrant

**DOI:** 10.1155/2014/242504

**Published:** 2014-05-08

**Authors:** Lovleen Kaur, Rajni Bala, Neha Kanojia, Manju Nagpal, Gitika Arora Dhingra

**Affiliations:** ^1^Chitkara College of Pharmacy, Chandigarh-Patiala National Highway, Rajpura, Patiala, Punjab 140401, India; ^2^NCRD's Sterling Institute of Pharmacy, Nerul, Navi Mumbai 400706, India

## Abstract

The current research work involves preparation of fast dissolving tablets of Aceclofenac by direct compression method using different concentrations of *Lepidium sativum* mucilage as natural superdisintegrant. A two-factor three-level (3^2^) factorial design is being used to optimize the formulation. Nine formulation batches (D1–D9) were prepared accordingly. Two factors as independent variables (*X*_1_-amount of *β*-cyclodextrin and *X*_2_-amount of *Lepidium sativum* mucilage) were taken with three levels (+1, 0, −1). The levels of two factors were selected on the basis of preliminary experiments conducted and their effect on three dependent variables (disintegration time, wetting time, and *in vitro* drug release) was studied along with their % prediction error. All the active blends were evaluated for postcompression parameters (angle of repose, Carr's index, Hausner ratio, etc.) and the tablets were evaluated for postcompression parameters (weight variation, hardness, and friability, wetting time, disintegration time, water absorption ratio, and *in vitro* drug release studies). The optimum batch was further used for SEM and stability studies. Formulation D5 was selected by the Design-Expert software which exhibited DT (15.5 sec), WT (18.94 sec), and *in vitro* drug release (100%) within 15 minutes.

## 1. Introduction

Oral route of drug administration has wide acceptance up to 50–60% of total people experience inconvenience in swallowing conventional dosage forms such as tablets when water is not available, in the case of motion sickness (kinetosis) and sudden episodes of coughing during the common cold, allergic conditions, and bronchitis. For these reasons, tablets that can rapidly dissolve or disintegrate in the oral cavity have attracted a great deal of attention. The development of orodispersible tablet has been vastly increased in the last few decades especially for the geriatric patients. Orodispersible tablets are not only indicated for people who have swallowing difficulties, but also are ideal for active people. Orodispersible tablets are also called mouth-dissolving tablets, melt-in-mouth tablets, fast dissolving tablets, rapidmelts, porous tablets, quick dissolving, and so forth [1, 2]. Orodispersible tablets are those when put on tongue disintegrates instantaneously, releasing the drug, which dissolves or disperses in the saliva. The faster the drug release into solution, the quicker the absorption and onset of clinical effect. The advantages of mouth dissolving dosage forms are increasingly being recognized in both industry and academics. Their growing importance was underlined recently when European Pharmacopoeia adopted the term “Orodispersible tablet” as a tablet to be placed in the mouth where it disperses rapidly before swallowing. The increasing popularity of orally disintegrating dosage forms is in part owing to various factors such as fast disintegration, good mouth feel, easy to handle, easy to swallow, and effective taste [3, 4].

Aceclofenac is a nonsteroidal agent with marked anti-inflammatory and analgesic properties. The mode of action of Aceclofenac is largely based on the inhibition of prostaglandin synthesis. Aceclofenac is a potent inhibitor of the enzyme cyclooxygenase, which is involved in the production of prostaglandins [5, 6]. Aceclofenac can be administered twice daily as 100 mg orally in the treatment of rheumatoid arthritis. Geriatric patients may have difficulty in swallowing and chewing the tablets resulting in patient noncompliance and ineffective therapy [7, 8]. To overcome these problems mouth dissolving tablets are a good option. Since, they disintegrate and dissolve rapidly in saliva without need for drinking water. The development of a fast dissolving tablet also provides an opportunity for a line extension in the market place.

## 2. Materials and Methods

### 2.1. Materials

Aceclofenac was obtained as a gift sample From Ind Swift Pvt. Ltd., Dera Bassi, India, sample.* Lepidium sativum* was procured from local market, Patiala, India. MCC from Ontop Pharmaceutical Pvt. Ltd., Bangalore, India, Aspartame from Ipza pharmaceutical Pvt. Ltd. Patiala, India, and *β*-Cyclodextrin from signet chemical corporation, Mumbai. All chemicals used were of analytical grade.

### 2.2. Methods

#### 2.2.1. UV Absorption Spectroscopy

*λ*_max⁡_ of the drug was determined by UV spectroscopy. Stock solution of drug was prepared by dissolving Aceclofenac in pH 6.8 phosphate buffer. 100 mg of drug was weighed and transferred to a 100 mL volumetric flask. The volume was made up to the mark with pH 6.8 phosphate buffer to obtain stock solution (A) having concentration of 1000 *μ*g/mL·1 mL of stock solution A was further diluted to 100 mL with pH 6.8 phosphate buffer to obtain stock solution (B) having concentration 10 *μ*g/mL. Aliquots of stock solution (B) was serially diluted to obtain solutions in concentration of 2 to 20 *μ*g/mL of drug with pH 6.8 phosphate buffer. The absorbance of the final solutions was measured at 275 nm spectrophotometer Systronics AU-2701, Mumbai, India.

#### 2.2.2. Fourier Transform Infrared Spectroscopy (FTIR)

FTIR spectrum was recorded of pure drug, polymer (Lepidium sativum mucilage), and mixture of drug with polymer. The samples were analysed by KBr pellet method using FTIR spectroscopy. About 10 mg of the formulation is mixed with dried potassium bromide of equal weight. The mixture is properly grinded using pestle and mortar. Pellets are formed by compressing the mixture using hydraulic press. Transparent pellets formed in this way were scanned. The spectra were scanned over a frequency range 4000–400 cm^−1^.

#### 2.2.3. Isolation of Mucilage from Lepidium Sativum

The seeds of* Lepidium sativum* contain the mucilage around the outer layer. The seeds were boiled with distilled water for 15 min and the mass was filtered through Buckner funnel without filter paper and the retained residue was boiled with distilled water for 15 min and the combined liquid was passed through eight folds of muslin cloth. Then the mucilage was precipitated from the filtrate by adding ethanol. The precipitated mucilage was dried in oven at 45°C till it was completely dried [9]. The dry powder was passed through 80 mesh sieve and stored in dissector for further evaluation.

#### 2.2.4. Determination of Swelling Index

A fine seed material (1 g) was placed into a 25 mL glass Stoppard measuring cylinder. The internal diameter of the cylinder was 16 mm and the length of the graduated portion was approximately 125 mm, marked in 0.2 mL divisions from 0 to 25 mL in an upward direction. 25 mL of water was added into the cylinder containing material and mixture was shaken thoroughly at interval of every 10 min for 1 h. Sample was allowed to stand for 3 h at room temperature and volume occupied by the plant material, including any sticky mucilage, was measured. The mean value was calculated, related to 1 g of plant material [10].

#### 2.2.5. Preparation of Fast Dissolving Tablets by Direct Compression Method

Fast dissolving tablets of Aceclofenac were prepared by direct compression method. All ingredients were mixed step by step then passed thorough sieve (number 100) and mixed with drug for 15 min in poly bag. Lubricants such as talc and magnesium stearate were added in this powder mixture at last and again mixed for 5 min. The active blends were compressed into tablets (300 mg) using single punch tablet machine. On the basis of results of preliminary batches, final formulation batches were prepared by using 3^2^ factorial design using two independent variables *X*_1_ and *X*_2_ where *X*_1_ is the amount of *β*-CD and *X*_2_ is the amount of* Lepidium sativum* mucilage. Three levels for each factor were selected (−1, 0, +1) indicating low, centre, and high values. Nine formulation batches of fast dissolving tablets were prepared and evaluated for precompression and postcompression parameters. The design layout and composition of 3^2^ factorial designs of Aceclofenac fast dissolving tablet formulation are given in Tables 1 and 2, respectively.

#### 2.2.6. Precompression Parameters

All formulation batches were evaluated for precompression parameters such as angle of repose, bulk density, tapped density, Carr's consolidation index, and Hausner's ratio as per the official methods [11].

#### 2.2.7. Postcompression Parameters

*Weight Variation*. The weight variation test is carried out in order to ensure uniformity in the weight of tablets in a batch. The total weight of 20 tablets from each formulation was determined and the average weight was calculated. The individual weight of the tablets was also determined accurately and the weight variation was calculated as specified in IP.

*Thickness.* Thickness of tablet was measured by using Vernier Calipers. Three tables were selected at random from each batch and average measurement of three readings was taken.

*Diameter*. Diameter of tablet was measured using Vernier Calipers. Three tablets were selected at random from each batch and average measurement of three readings was taken.

*Hardness.* Hardness or crushing strength is the force required to break a tablet in a diametric compression was measured using Monsanto tablet hardness tester. It is expressed in kg/cm^2^.

*Friability Test.* Friability test was carried out using Roche Friabilator. 20 tablets from each formulation were weighed and placed in Roche Friabilator rotated at 25 rpm for 4 minutes. The tablets were dedusted and weighed again [12]. The percentage of weight loss was calculated using the formula

(1) $$\begin{array}{c} \%\text{Friability}=\;\frac{\left[\left(W_{1}-W_{2}\right)100\right]}{W_{1}}\;, \end{array}$$

where *W*_1_ is the weight of tablet before test and *W*_2_ is the weight of tablet after test.

*Wetting Time.* Five circular tissue papers of 10 cm diameter were placed in a Petri dish (10 cm diameter). Ten mL of water containing eosin, a water soluble dye, was added to Petri dish. A tablet was carefully placed on the surface of the tissue paper. The time required for water to reach upper surface of the tablet is noted as wetting time [13].

*Water Absorption Ratio.* A piece of tissue paper folded twice was placed in a small Petri dish (6 mm) diameter containing 6 mL of water. A tablet was put on the paper and the time required for complete wetting was measured [14]. The wetted tablet was then weighed. Water absorption ratio, *R*, was determined using following equation:

(2) $$\begin{array}{c} R=100\times \frac{\left(W_{a}-W_{b}\right)}{W_{b}}, \end{array}$$

where *W*_*b*_ is the weight of tablet before water absorption and *W*_*a*_ is the weight of tablet after water absorption. 

*In Vitro Disintegration Test*. The USP disintegration test apparatus was used to determine disintegration time. Six tablets from each formulation were tested in 900 mL of water at 37°C. The study was done in triplicate.

*In Vitro Drug Release Studies. In vitro *drug release was carried using USP II dissolution apparatus. The formulations were subjected to* in vitro *dissolution studies by using 900 mL of pH 6.8 phosphate buffers kept at 37 ± 0.5°C at a speed 50 rpm. The aliquots were collected at specified time intervals (5, 10, 15, 30, and 45 min) and analyzed at 275 nm by UV Spectrophotometer Systronics AU-2701, Mumbai, India. Cumulative drug release was then calculated. The study was done in triplicate.

*Scanning Electron Microscopy (SEM).* The surface characteristics of optimum tablet formulation were evaluated by SEM studies. The micrographs were recorded using scanning electron microscope (JEOL JSM-6100**)**. The samples were mounted on a double-sided tape on aluminium stubs and were sputter coated with gold using fine coat ion sputter (JEOL).

#### 2.2.8. Factorial Design

A statistical model incorporating interactive and polynomial terms was used to evaluate the responses:

(3) $$\begin{array}{c} Y=b_{0}+b_{1}X_{1}+b_{2}X_{2}+b_{12}X_{1}X_{2}+b_{11}X_{21}+b_{22}X_{22}, \end{array}$$

where *Y* is the dependent variable and *b*_0_ is the arithmetic mean response of the 9 runs. The main effects (*X*_1_ and* X*_2_) represent the average result of changing one factor at a time from its low to high value. The interaction terms (*X*_1_ *X*_2_) show how the response changes when 2 factors are simultaneously changed. The polynomial terms (*X*_21_ and *X*_22_) are included to investigate nonlinearity [15, 16].

#### 2.2.9. Accelerated Stability Studies

The stability studies of selected tablet batches were carried out in stability chamber (Remi Instruments, India) kept at 40°C and 75% RH conditions for three months. The effects of temperature and time on the physical characteristics of the tablet were evaluated for assessing the stability of the prepared formulations. The tablets were evaluated for their physicochemical parameters (such as hardness, thickness, diameter, friability,* in vitro *disintegration time, wetting time, drug content, and* in vitro *dissolution) after 15 days, 1 month, 2 month, and 3 months.

## 3. Results and Discussions

### 3.1. FTIR (Fourier Transform Infrared Spectroscopy)

**Figure 1:**
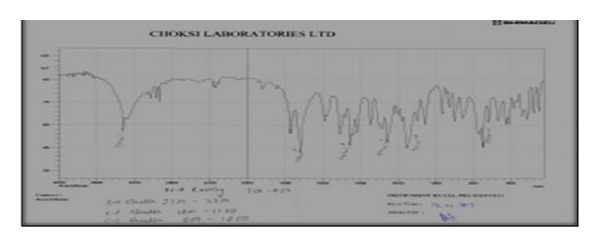
FTIR spectra of pure drug (Aceclofenac).

**Figure 2:**
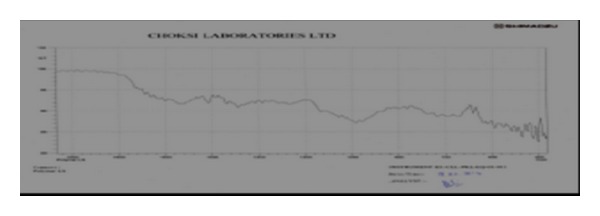
FTIR spectra of* Lepidium sativum* mucilage.

**Figure 3:**
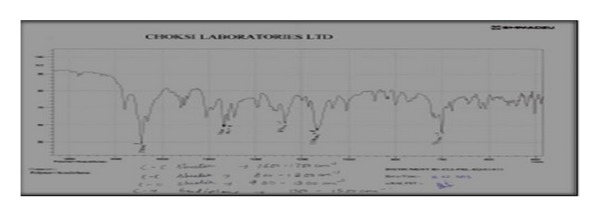
FTIR spectra of mixture of drug and mucilage.

IR spectra of pure drug,* Lepidium sativum* mucilage, and mixture of drug with mucilage are shown in Figures 1, 2, and 3, respectively. The prominent IR absorption peaks of Aceclofenac showed at 3319 and 3267 that these broad peaks may be due to OH hydrogen bonding. 2970 is NH aromatic stretching; peaks near 2937 including 1921 may be due to CH stretching of CH2 groups, carbonyl group vibration at 1770 and 1716. Peaks at 1589, 1577, and 1508 indicate the presence of C=C ring stretching. The IR spectra of polymer indicated amorphous nature of polymer. Presence of all the characteristics peaks of drug in IR spectra of drug polymer mixture indicates no interaction between drug and carrier.

### 3.2. Swelling Index

The swelling ratio of mucilage, determined in distilled water, was observed to be 4.1 ± 0.6. There was a significant change in swelling by the end of the study, which indicated that the mucilage had excellent swelling properties.

### 3.3. Precompression Parameters

The results of precompression studies (bulk density, tapped density, angle of repose, Carr's index, etc.) of final active blends (D1–D9) are given in Table 3. The results of bulk density and tapped density ranged from 0.458 ± 0.021 to 0.515 ± 0.041 and 0.548 ± 0.023 to 0.605 ± 0.040, respectively. The results of angle of repose (20.30 ± 0.21 to 25.64 ± 0.25) indicated good flow properties which were further supported by Carr's index (11.7 to 17.6) and Hausner's ratio data (1.13 to 1.21).

### 3.4. Postcompression Parameters

The results of postcompression studies are depicted in Table 4. All nine formulations were uniform in dimensions and exhibited sufficient hardness in the range from 2.0 ± 0.3 to 3.0 ± 0.4. The friability data (<1%) indicated sufficient resistance to abrasion. All the formulation batches passed weight variation test. The disintegration values less than 1 min were observed in all formulations. The superdisintegrant (mucilage) alone has a significant impact on disintegration and wetting characteristics as seen in batches D1, D4, and D7. This may be due to enhanced swelling characteristics due to presence of mucilage [17]. However the disintegration characteristics are not favored at highest concentrations of superdisintegrant (mucilage) and *β*-CD. The addition of *β*-CD in batches D2, D5, and D8 also improved the disintegration characteristics but not significantly as the superdisintegrant. Higher amounts of *β*-CD in batches D3, D6, and D9 retard the disintegration as observed by increased DT and WT values and therefore drug release was also retarded. *β*-CD was added to enhance the dissolution characteristics which were observed in batch D5 (i.e., 100% drug release was observed within 30 min). D5 batch was observed as promising batch based upon disintegration and dissolution data.

### 3.5. *In Vitro* Drug Release Studies

**Figure 4:**
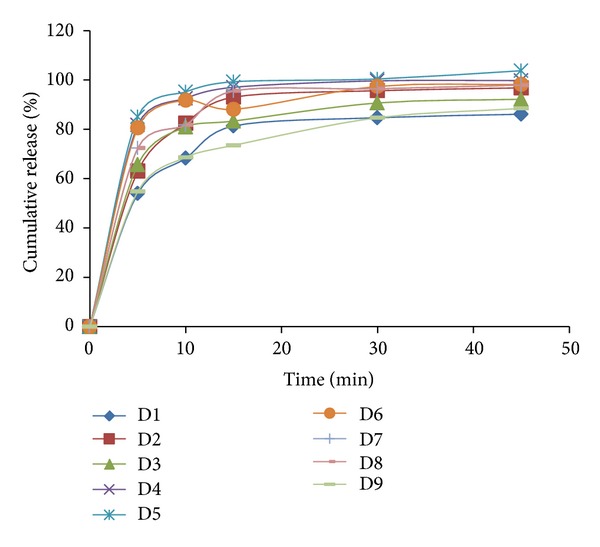
*In vitro *drug release profile of final batches (D1–D9).

*In vitro *drug release of all final formulation batches (D1–D9) is given in Figure 4. More than 50% drug release was observed within 5 min in all formulation batches. The increased concentration of mucilage leads to significantly enhanced drug release (D4–D6 batches as compared to D1–D3). However, the increase in drug release was not significant at highest levels of superdisintegrant. The addition of *β*-CD also improves dissolution of drug as seen in batches D1 and D2; batches D4 and D5; batches D7 and D8. However higher concentration of *β*-CD retard drug dissolution (in D3, D6, and D9) which may be due to viscous networks. All the formulations showed more than 80% drug release in 15 min except batch D9. D6 batch was observed as promising batch as per dissolution data (100% drug release in 30 min).

### 3.6. Factorial Design

#### 3.6.1. *In Vitro* Disintegration Time

The response surface plot demonstrated the effect of amount of *β*-CD and* Lepidium sativum* mucilage on disintegration time (DT). The polynomial equation indicated that disintegration time was decreased from 25 ± 2 to 19 ± 2 and from 26 ± 3 → 22 ± 2 → 30 ± 2 at low and high level of *β*-CD, respectively, as the concentration of the* Lepidium sativum* was increased. The DT value was changed from 25 ± 2 → 22 ± 2 → 26 ± 3 and from 19 ± 2 to 30 ± 2 at low and high levels of* Lepidium sativum*, respectively, as the concentration of *β*-CD was increased. Increased concentration of* Lepidium sativum* mucilage has significant effect on DT at low concentration of *β*-CD, whereas it has a negative effect on DT at high concentration of *β*-CD. Increased concentration of *β*-CD exhibited a random effect on DT value at low level of* Lepidium sativum* mucilage and a significant negative effect (increase in DT value) at high level of* Lepidium sativum* mucilage.

The parameter disintegration time can be described by the following model equation:

(4) DT=+15.89+2.33∗X1−0.67∗X2+2.50∗X1∗X2 +4.67∗X12+4.67∗X22.

#### 3.6.2. Wetting Time

The response surface plot demonstrated the effect of amount of *β*-CD mucilage and* Lepidium sativum* mucilage on wetting time (WT). The polynomial equation indicated that wetting time was decreased from 29 ± 3 to 21 ± 2 and followed a random order (i.e., from 31 ± 3 → 28 ± 2 → 38 ± 1) at low and high level of *β*-CD, respectively, as the concentration of the* Lepidium sativum* was increased. The wetting time value was first decreased from 29 ± 3 to 26 ± 2 and increased from 26 ± 2 to 31 ± 3 at low level of* Lepidium sativum* mucilage (with increasing amount of *β*-CD) and increased from 21 ± 2 to 38 ± 1 at high level of* Lepidium sativum* mucilage, as the concentration of *β*-CD was increased. Increased concentration of *β*-CD exhibited a random effect on WT (even negative effect at higher concentration). Increased concentration of* Lepidium sativum* mucilage favours wetting characteristics at low *β*-CD, whereas the effect was reversed at highest *β*-CD concentration.

The parameter wetting time can be described by the following model equation:

(5)   WT=+19.67+3.833∗X1−0.33∗X2+3.75∗X1 +5.5∗X12+5.00∗X22.

#### 3.6.3. *In Vitro* Drug Release

The response surface plot demonstrated the effect of amount of *β*-CD and* Lepidium sativum* mucilage on* in vitro *drug release. The polynomial equation indicated that drug release was increased from 81.33% to 91.05% and from 83.33% → 88.05% → 73.49% at both low and high levels of *β*-CD with increasing concentration of* Lepidium sativum*. The drug release was first increased and then decreased, that is, 81.33% → 93.02% → 83.33% at low level of* Lepidium sativum* with increasing concentration of *β*-CD. At high level of* Lepidium sativum* with increasing concentration of *β*-CD, the drug release follows random order, that is, 91.05% → 95.52% → 73.49%. Increased concentration of* Lepidium sativum* mucilage has significant effect on* in vitro *release. *β*-CD showed random and negative effect on drug release.

The parameter* in vitro* drug release can be described by the following model equation:

(6) DR(15 min⁡) =+99.93−2.84∗X1+0.40∗X2 −4.89∗X1∗X2−11.47∗X12−6.0∗X22.

Various response parameters and summary of regression analysis and ANOVA are shown in Tables 5 and 6, respectively. 3D RSM plot for disintegration time, wetting time, and* in vitro* drug release is shown in Figure 7.

#### 3.6.4. Numerical Optimization

A numerical optimization technique using the desirability approach as shown in Figures 8 and 9 was employed to develop a new formulation with the desired responses. Upon the evaluation, the formulation composition with *β*-CD concentration and the amount of* Lepidium sativum* mucilage which fulfilled maximum requirements of an optimum formulation with desirability of 0.9713 was observed. The optimized formulation was evaluated for various dependent variables. The response variables were calculated and compared to the corresponding predicted values.  Table 7 shows the observed and predicted response values along with % prediction errors. Over lay plot showing three optimized dependent variables is given in Figure 10.

### 3.7. Scanning Electron Microscopy

**Figure 5:**
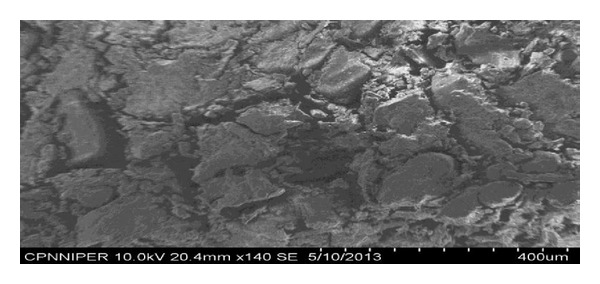
SEM image for optimised batch D5.

The SEM photographs of optimised batch D5 are shown in Figure 5. Porous tablet surface was observed which may be due to the presence of superdisintegrant.

### 3.8. Accelerated Stability Studies

**Figure 6:**
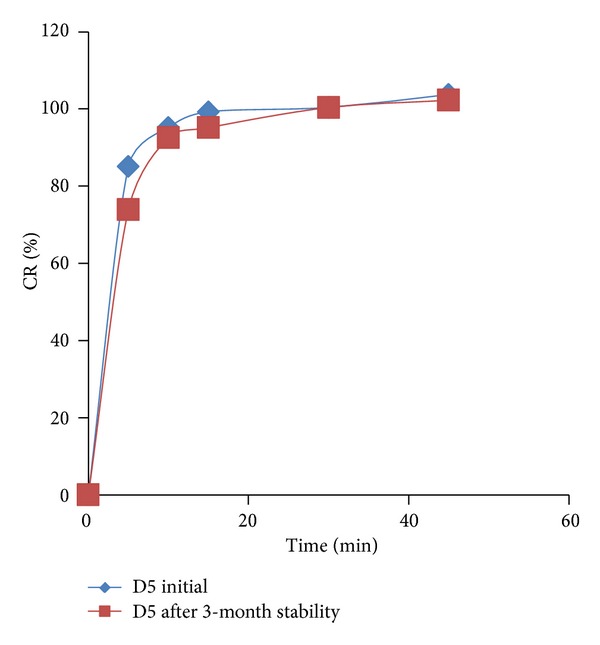
Comparison of dissolution profile of batch D5 before stability and after stability.

**Figure 7:**
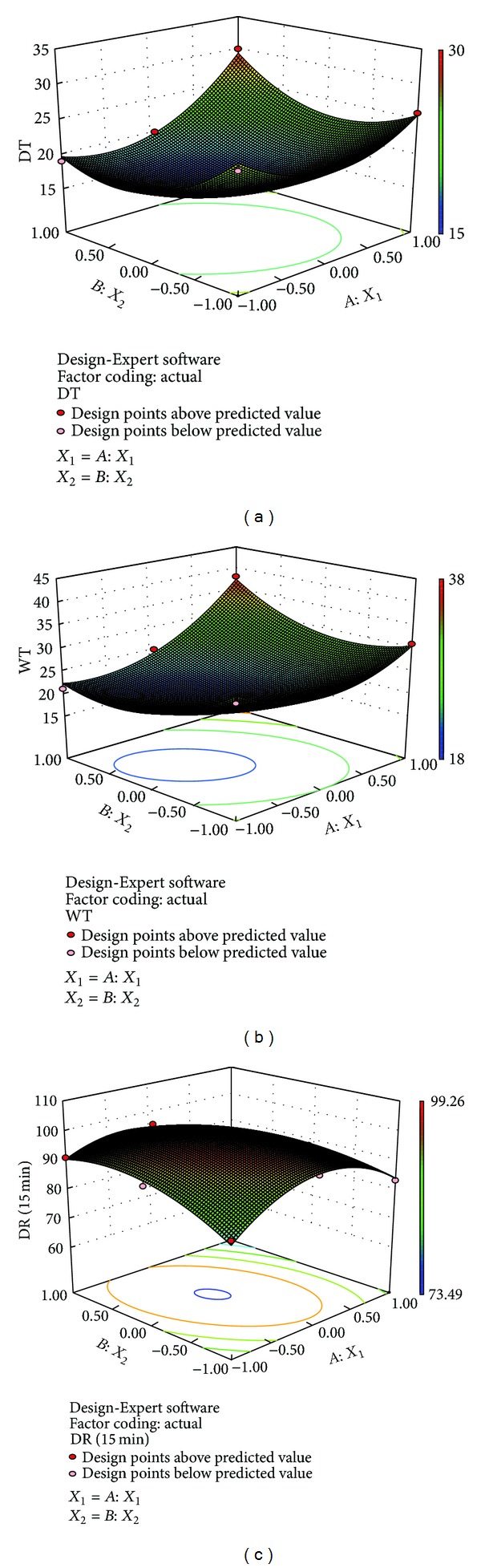
3D RSM plots for (a) DT, (b) WT, and (c) drug release (15 min).

**Figure 8:**
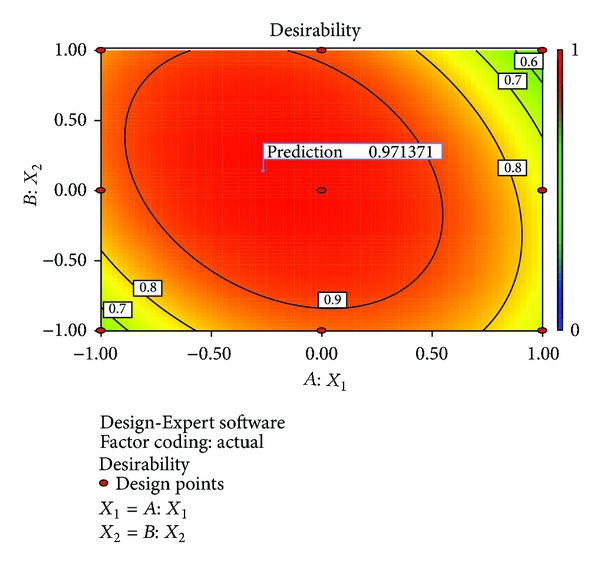
Desirability plot (2D).

**Figure 9:**
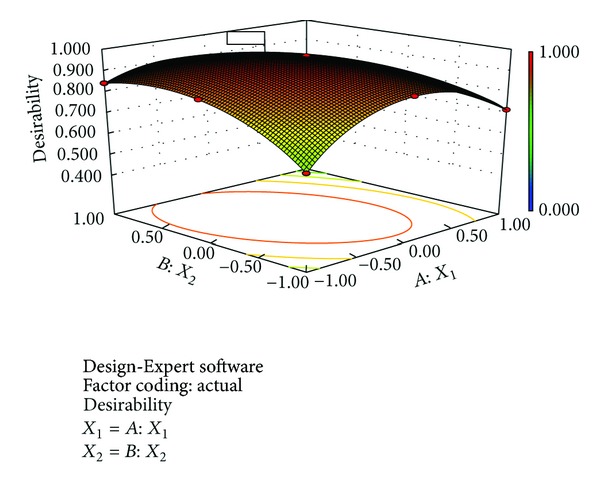
Desirability plot (3D).

**Figure 10:**
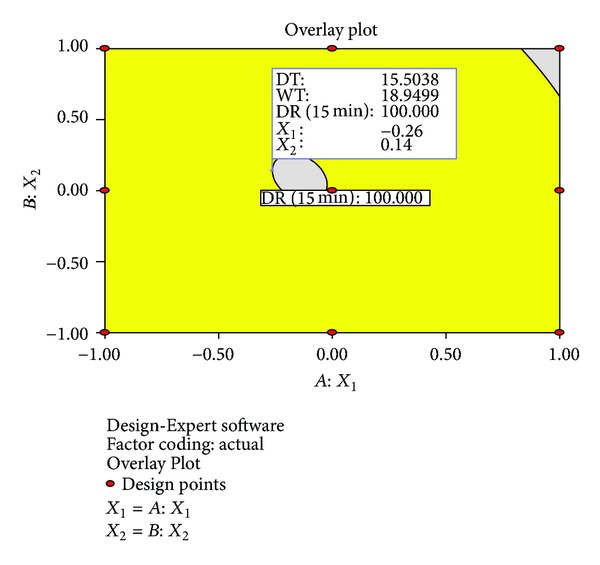
Overlay plot showing three optimized dependent variables.

Stability studies were carried out using optimum batch D5 as per ICH guidelines for 90 days at accelerated stability condition (40°C/75% RH). No remarkable changes were observed in batch D5 (physicochemical properties as well as release profile) as shown in Table 8. This indicates good stability of the formulation even after stressed conditions.* In vitro *drug release studies were also done after 3-month stability testing and compared with initial D5 batch (before stability) both drug release curves are paralleled indicating no change in drug release behaviour for D5 tablet after 3 months of stability testing as shown in Figure 6.

## 4. Conclusion

Fast dissolving tablets of Aceclofenac were formulated and optimized using 3^2^ factorial design. Two independent variables, that is, amount of *β*-cyclodextrin and amount of* Lepidium sativum *mucilage at three levels were selected on the basis of preliminary studies. Addition of superdisintegrant* Lepidium sativum* mucilage leads to significant effect on disintegration characteristics as well as drug release. But higher concentrations of mucilage had negative impact on drug release and disintegration. Addition of *β*-cyclodextrin leads to improved dissolution characteristics not much affecting disintegration time but higher concentration of *β*-CD retard drug disintegration and drug release. The porous nature of tablet with swelling and wicking characteristics of mucilage along with increased drug solubility by *β*-CD in combination lead to maximum drug release from fast dissolving tablets. Design-Expert software was used to optimize and response surface plots and contour plots were drawn, and optimum formulations were selected by feasibility and grid searches. Polynomial mathematical models, generated for various response variables using multiple regression analysis, were found to be statistically significant (*P* < 0.05). Formulation D5 was selected by the Design-Expert software which exhibited DT (15.5 sec), WT (18.94 sec), and* in vitro *drug release (100%) within 15 minutes.

## Figures and Tables

**Table 1:** Design layout of 3^2^ factorial design.

Formulation batches	*X*_1_	*X*_2_
D1	−1	−1
D2	0	−1
D3	+1	−1
D4	−1	0
D5	0	0
D6	+1	0
D7	−1	+1
D8	0	+1
D9	+1	+1

Where 1 is the high value, −1 is the low value, and 0 is the centre value for the factors X_1_ and X_2_.

X_1_: amount of *β*-cyclodextrine.

X_2_: amount of *Lepidium sativum* mucilage.

**Table 2:** Composition of 3^2^ factorial design batches of Aceclofenac fast dissolving tablets.

Ingredients (mg)	D1	D2	D3	D4	D5	D6	D7	D8	D9
Aceclofenac	100	100	100	100	100	100	100	100	100
*β*-CD	0	15	30	0	15	30	0	15	30
*Lepidium Sativum* Mucilage	6	6	6	15	15	15	24	24	24
MCC	144	129	114	135	120	105	126	111	96
Mannitol	30	30	30	30	30	30	30	30	30
Aspartame	10	10	10	10	10	10	10	10	10
Talc	4	4	4	4	4	4	4	4	4
Magnesium Stearate	6	6	6	6	6	6	6	6	6

MCC: microcrystalline cellulose, *β*-CD: *β*-cyclodextrine.

**Table 3:** Evaluation of precompression parameters of factorial design batches of Aceclofenac fast dissolving tablets.

Parameter	D1	D2	D3	D4	D5	D6	D7	D8	D9
Bulk density (g/mL)	0.481 ± 0.023	0.484 ± 0.014	0.458 ± 0.021	0.514 ± 0.040	0.515 ± 0.041	0.504 ± 0.029	0.482 ± 0.025	0.484 ± 0.043	0.514 ± 0.009
Tapped density (g/mL)	0.584 ± 0.013	0.548 ± 0.023	0.550 ± 0.020	0.587 ± 0.009	0.589 ± 0.018	0.590 ± 0.021	0.570 ± 0.015	0.556 ± 0.019	0.605 ± 0.040
Angle of repose	20.30 ± 0.21	24.70 ± 0.13	21.30 ± 0.25	22.78 ± 0.32	22.79 ± 0.08	21.30 ± 0.15	22.29 ± 0.22	23.74 ± 0.09	25.64 ± 0.25
Carr's index	17.6	11.7	16.6	12.5	12.8	14.06	15.2	12.9	15.0
Hausner ratio	1.21	1.13	1.20	1.14	1.14	1.17	1.18	1.14	1.17

Data are represented as mean ± SD (*n* = 3).

**Table 4:** Evaluation of postcompression parameters of factorial design batches of Aceclofenac fast dissolving tablets.

Parameters	D1	D2	D3	D4	D5	D6	D7	D8	D9
Hardness (kg/cm^2^)	2.0	2.5	3.0	2.0	2.5	3.0	2.0	2.5	3.0
Friability (%)	0.9	0.62	0.31	0.9	0.65	0.33	0.9	0.68	0.38
Disintegration time (sec)	22	25	26	20	15	22	19	20	30
*In vitro* dispersion time (sec)	31	24	38	24	21	29	20	36	34
Wetting time (sec)	29	26	31	24	18	28	21	25	38
Water absorption ratio (%)	91.7	131	132	126	134	135	128	129	122

Data are represented as mean ± SD (*n* = 3).

**Table 5:** Response parameters of various formulations.

Batches	*X*_1_ (amount of *β*-CD)	*X*_2_ (amount of *Lepidium sativum* mucilage)	DT (sec) (*R*1)	WT (*R*2)	% cumulative release (*R*3)
F1	−1	−1	25	29	81.33
F2	0	−1	20	24	93.02
F3	+1	−1	19	21	83.33
F4	−1	0	22	26	89.55
F5	0	0	15	18	99.26
F6	+1	0	20	25	88.05
F7	−1	+1	26	31	91.05
F8	0	+1	22	28	95.52
F9	+1	+1	30	38	73.49

Data are represented as mean ± SD (*n* = 3).

**Table 6:** Summary of results of regression analysis and ANOVA for measured response.

Response	*B*0	*B*1	*B*2	*B*11	*B*12	*B*22
DT						
Full model	15.89	2.33	−0.666	2.50	4.666	4.666
*P* value	0.0342	0.036	0.376	0.0503	0.0248	0.0248
Regression	DF = 5	SS = 147.44	MS = 29.49	*F* = 11.88	*R* ^2^ = 0.9519	
WT						
Full model	19.67	3.83	−0.33	3.75	5.5	5.00
*P* value	0.0476	0.0278	0.7501	0.491	0.0449	0.0566
Regression	DF = 5	SS = 255.58	MS = 51.12	*F* = 9.34	*R* ^2^ = 0.9396	
T15						
Full model	99.93	−2.48	0.40	−4.89	−11.47	−6.00
*P* value	0.0197	0.0584	0.7052	0.0248	0.0061	0.0359
Regression	DF = 5	SS = 479.99	MS = 96.00	*F* = 17.62	*R* ^2^ = 0.9671	

**Table 7:** Comparison of observed and predicted value with the prediction error.

Response parameters	Constraints set	Predicted value	Observed value	% prediction error
Disintegration time	Minimize	15.50	16.33	5.08
Wetting time	Minimize	18.94	18.33	3.32
*In vitro* drug release	Maximize	100.00	99.66	0.34

**Table 8:** Stability studies of optimized batch D5 at accelerated condition.

Time	Parameter						
	Weight variation	Tablet Thickness (mm)	Tablet diameter (mm)	Hardness (kg/cm^2^)	Wetting time (sec)	Friability (%)	Disintegration time (sec)
15 days	297.49	3.36 ± 0.04	8.07 ± 0.02	2.51 ± 0.3	20 ± 2	0.65 ± 0.04	14 ± 2
30 days	297.51	3.38 ± 0.04	8.08 ± 0.02	2.47 ± 0.3	19 ± 2	0.67 ± 0.03	15 ± 2
60 days	297.76	3.41 ± 0.03	8.14 ± 0.02	2.45 ± 0.3	19 ± 2	0.61 ± 0.03	14 ± 2
90 days	300.00	3.43 ± 0.04	8.14 ± 0.02	2.49 ± 0.3	18 ± 2	0.63 ± 0.03	14 ± 2
